# Hypotheses relating to the function of the claustrum II: does the claustrum use frequency codes?

**DOI:** 10.3389/fnint.2014.00007

**Published:** 2014-01-29

**Authors:** John Smythies, Lawrence Edelstein, Vilayanur Ramachandran

**Affiliations:** ^1^Department of Psychology, Center for Brain and Cognition, University of California San DiegoSan Diego, CA, USA; ^2^Medimark CorporationDel Mar, CA, USA

**Keywords:** claustrum, spectral processing, synchronized oscillations, concatenation, frequency code

## Introduction

In previous papers (Smythies et al., 2012, 2014) we presented the general hypothesis that the claustrum may be concerned with information processing operations on synchronized gamma oscillations in the brain at three levels. At the first level (subhypothesis 1) it just magnifies the oscillations in cortico-claustral circuits. At the second level (subhypothesis 2) it may integrate these oscillations. At the third level (subhypothesis 3) it might process the contained spike codes. These previous papers were concentrated upon the first level. In this paper we will review arguments that both subhypotheses 2 and 3 are, at present, unsatisfactory. Subhypothesis 1, however, remains satisfactory.

The claustrum consists of a body of densely interconnected P (pyramidal) cells and GABAergic interneurons (INs) arranged in a sheet of gray matter underlying the insula in the basal telencephalon of the mammalian brain. These cells are arranged in functional units, each of which is connected with a particular cortical or subcortical area with which the claustrum maintains reciprocal relationships, which encompass practically every such area. There is evidence that it is particularly concerned with salient activities requiring integration between two or more of these areas [see Smythies et al. (2012, 2014), for details]. One structurally facilitative feature of this anatomy is that the claustrum has a number of afferents/efferents along a topographical spectrum. There are well-defined arrays of visual-, auditory-, and somatosensory-associated zones (or “maps”), plus extensive limbic connections with the ventral claustrum. To the extent studied, individual P cells have been shown to receive a (mostly) modality-specific input. For example, visual zone neurons respond (almost) exclusively to visual stimuli, auditory neurons respond (almost) exclusively to auditory stimuli, etc. (Remedios et al., 2010). The afferent glutamatergic axons of cortical layer VI P cells densely synapse on claustral P cells. In turn, these claustral P cells reciprocate by sending efferents back to the very same cortical area from which their afferent input originated. It has been demonstrated that some claustral P cells have bifurcated axonal projections to different cortical areas. Of particular significance is that claustral P cells also maintain direct contact with claustral GABAergic INs via local collaterals forming a dense axonal array. The INs quite likely form an interactive gap-junction syncytium.

This structure entails that the afferent inflow to the claustrum will carry multiple impulses with different power spectra and synchronized at different frequencies that will have ample opportunity to interact in complex ways within the densely packed amorphous syncytium that constitutes the interior of the claustrum. The results of these interactions, in the form of processed and integrated information, might then be transported to selected areas of the brain via the efferent network. The question that we will address now is what is the validity of subhypotheses 2 and 3.

## Interactions between nets oscillating at different frequencies

There are several ways how neuronal synchrony may support the encoding of information about stimuli. The most extensively investigated is the modulation of the strength of synchronization. This would come under the aegis of our hypothesis level 1. Another utilizes the delays or phase-offsets among the discharges of neurons that are participating in synchronized assemblies (Uhlhaas et al., 2009). Recently attention has been paid to the integration of two assemblies with synchronized oscillations at two different frequencies. These authors continue,

“Remarkably, synchronization is not confined to oscillations of the same frequency band but occurs across different frequencies as *n*:*m* synchrony This allows for the concatenation of rhythms and for the establishment of partial correlations. An attractive hypothesis is that this could serve as a mechanism to encode nested relations – an indispensable function for the neuronal representation of composite objects and movements.”

Palva et al. (2005) demonstrated, using magnetoencephalography, that robust cross-frequency phase synchrony is present in the human cortex among oscillations with frequencies from 3 to 80 Hz, and is modulated by cognitive tasks. Other examples of cross-frequency interactions are detailed by Wang and Delgutte (2012), Fitzgerald et al. (2013), Herman et al. (2013).

A comprehensive review of nested synchrony is presented by Monto (2012).

Another possible mechanism is provided by the finding that a relatively broad range of spatiotemporally structured input patterns are translated into specific output patterns in hippocampal CA3 neuron dendrites mainly by a mechanism involving a G protein activated inwardly rectifying current (GIRK) (Makara and Magee, 2013). If this mechanism is also found in the claustrum that might provide the mechanism we are looking for. The integrating mechanism here would operate in the dendrites of claustral PV + INs that receive input from collaterals of different modalities of claustral P cell outputs.

## Spectral processing and concatenation

Could the claustrum operate by concatenation and spectral processing? Roopun et al. (2008) showed that two co-active local circuits can combine sequentially to generate a third frequency whose period is the concatenation sum of the original two (see Figure 5 in this paper). These authors suggest that this may constitute a robust mechanism for combining information processed on multiple concurrent spatiotemporal scales. Significantly they add that concatenation of two different frequencies is possible for any given pair of rhythms and that these interactions between frequency bands may be as important as the individual frequencies themselves—a phenomenon termed spectral processing.

Different areas of the brain have different types of synchronized gamma oscillations. Concatenation has been detected using *in vitro* specimens of rat somatosensory cortex by Kramer et al. (2008). Initially separate synchronous gamma (25 ms period) and beta rhythms (40 ms period), in the superficial and deep layers of the cortex respectively, underwent a concatenative transition to a synchronous beta rhythm (65 ms period) in all cortical layers. In rat primary cortex a 30–45 Hz gap-junction-dependent gamma rhythm dominates rhythmic activity in supragranular layers 2/3 whereas a depolarization dependent 50–80 Hz rhythm is expressed in granular layer 4 (Ainsworth et al., 2011). Interestingly spectrally identical patterns of beta2 and gamma rhythms can be generated in primary sensory areas and polymodal association areas by fundamentally different local circuit mechanisms: e.g., glutamatergic excitation induced beta2 frequency population rhythms only in layer 5 association cortex; whereas cholinergic neuromodulation induced this rhythm only in layer 5 primary sensory cortex (Roopun et al., 2008). NMDA receptor-dependent mEC gamma rhythms are mediated by basket interneurons, but NMDA receptor-independent gamma rhythms are mediated by a novel interneuron subtype-the goblet cell (Middleton et al., 2008).

## Coding aspects of spectral processing and dendritic integration in the claustrum

Is it possible that spectral processing, or dendritic integration, may occur in the claustrum (subhypothesis 2)? We have seen how the interior of the claustrum may be the scene of a dynamic process whereby grouped individual neurons, in close anatomical and functional contact, may continually modulate their oscillation patterns in response to a rapidly changing pattern of inputs. This might lead to the generation of new frequencies of oscillations each of which carries a unique code. For example, the hypothesis could be put forward that if a neuron (or group of neurons) receives two inputs, one oscillating at x Hz and the other at y Hz, and generates an oscillation in its output at a new frequency z, then z, whatever it is, could carry information that x has been bound to y. Translated into features of the stimuli that led to the production of x and y, this entails that z now carries information that x and y are “bound.” However, further consideration shows that this argument may have a basic fault. In any equation (a + b = c) there are many values of a and b that add to yield c. Therefore, one neuron in a feed forward chain, that receives an input oscillating at c Hz from another neuron, has no way of “telling” whether this represents a primary oscillation at c Hz, or which among the many values for a and b combinations that yield c. Thus, as an information processing system, it is useless.

Thus, in the case of any mechanism that “blends” the two incoming wave into a complex exit wave, the latter would have to be “interpreted” by a complicated decoding mechanism in the receiving neuron. This seems unnecessarily complex and rather pointless, in contrast to the simple mechanism proposed by hypothesis 1. However, as we cannot say at this instant that we *know* for certain that such an operation does not take place, we suggest that, although it may be too early to abandon subhypotheses 2 and 3 entirely, we should at least put them on the back burner.

Moreover, even if this were not so, concatenation and nesting may not be good models for the type of integration we are proposing. As one referee pointed out, the papers by Roopun et al. (2008) report a drift from gamma and beta2 to a more general interlaminar beta1 spectral frequency. However, consensus suggests that widespread high amplitude slowing of the EEG represents a loss of information, not a gain. Also, if concatenation already occurs at the level of even isolated primary sensory cortex (as the cited papers amply demonstrate), what role does the claustrum serve simply to project this information back to the original sources of information?

These arguments indicate down grading subhypothesis 2. The same argument applies to subhypothesis 3. In this the formula (a + b = c) applies to oscillatory activity of a population of spiking neurons. Furthermore, Gielen (2014) has argued that, if a neuron receives two inputs with spiking at different frequencies, it never responds by emitting spikes at a new frequency, but always chooses one or the other of the input frequencies. So this subhypothesis needs to be downgraded as well.

This leaves subhypothesis 1, which is not affected by these arguments and can be elevated to the status of a full hypothesis. In any case, this hypothesis seems preferable on the grounds of simplicity and adequacy. It suggests that what the claustrum does is only to magnify synchronized gamma oscillations, in conjunction with widespread cortical and subcortical areas, by means of the Pearson mechanism and the close packing of claustral neurons. In the case of neuronal assemblies that are working together, this promotes simultaneous multimodal processing by recruitment of different cortical and subcortical areas to work on the common task. In the case of competing neuronal assemblies (as when a decision or choice has to be made) the claustrum may provide the venue for a winner-takes-all competition between these assemblies that results in choice and behavior. This hypothesis can be viewed in the context of the well established and similar proposals put forward by Gray and McNaughton (2000) on how the hippocampus engages competitive interactions of neuronal assemblies to effect choices in goal directed behavior.

We feel it is important to down grade the more improbable subhypotheses 2 and 3 because hypotheses need eventually to be tested by experiment. We suggest that experiments to test only the more probable hypothesis 1 are presently indicated, unless and until new evidence to support its rivals is obtained.
